# Endocannabinoids, endocannabinoid-like molecules and their precursors in human small intestinal lumen and plasma: does diet affect them?

**DOI:** 10.1007/s00394-020-02398-8

**Published:** 2020-10-26

**Authors:** Silvia Tagliamonte, Chris I. R. Gill, L. Kirsty Pourshahidi, Mary M. Slevin, Ruth K. Price, Rosalia Ferracane, Roger Lawther, Gloria O’Connor, Paola Vitaglione

**Affiliations:** 1 Department of Agricultural Sciences, University of Naples “Federico II”, Via Università 100, 80055 Portici, NA Italy; 2 Nutrition Innovation Centre for Food and Health (NICHE), School of Biomedical Sciences, Ulster University, Coleraine, UK; 3 Altnagelvin Area Hospital, Western Health and Social Care Trust, Glenshane Road, Londonderry, UK

**Keywords:** Lipid mediators, Nutrient sensing, Ileal fluids, Gastrointestinal receptors, *N*-acylethanolamines, Ileostomists

## Abstract

**Purpose:**

To determine the small intestinal concentration of endocannabinoids (ECs), *N*-acylethanolamines (NAEs) and their precursors *N*-acylphosphatidylethanolamines (NAPEs) in humans. To identify relationships between those concentrations and habitual diet composition as well as individual inflammatory status.

**Methods:**

An observational study was performed involving 35 participants with an ileostomy (18W/17M, aged 18–70 years, BMI 17–40 kg/m^2^). Overnight fasting samples of ileal fluid and plasma were collected and ECs, NAEs and NAPEs concentrations were determined by LC-HRMS. Dietary data were estimated from self-reported 4-day food diaries.

**Results:**

Regarding ECs, *N*-arachidonoylethanolamide (AEA) was not detected in ileal fluids while 2-arachidonoylglycerol (2-AG) was identified in samples from two participants with a maximum concentration of 129.3 µg/mL. In contrast, mean plasma concentration of AEA was 2.1 ± 0.06 ng/mL and 2-AG was 4.9 ± 1.05 ng/mL. NAEs concentrations were in the range 0.72–17.6 µg/mL in ileal fluids and 0.014–0.039 µg/mL in plasma. NAPEs concentrations were in the range 0.3–71.5 µg/mL in ileal fluids and 0.19–1.24 µg/mL in plasma being more abundant in participants with obesity than normal weight and overweight. Significant correlations between the concentrations of AEA, OEA and LEA in biological fluids with habitual energy or fat intakes were identified. Plasma PEA positively correlated with serum C-reactive protein.

**Conclusion:**

We quantified ECs, NAEs and NAPEs in the intestinal lumen. Fat and energy intake may influence plasma and intestinal concentrations of these compounds. The luminal concentrations reported would allow modulation of the homeostatic control of food intake via activation of GPR119 receptors located on the gastro-intestinal mucosa.

**Clinical trial registry number and website:**

NCT04143139; www.clinicaltrials.gov.

**Electronic supplementary material:**

The online version of this article (10.1007/s00394-020-02398-8) contains supplementary material, which is available to authorized users.

## Introduction

Endocannabinoids (ECs) and their structural congeners *N*-acylethanolamines (NAEs), also known as “endocannabinoid-like molecules”, are endogenous lipid mediators involved in a wide range of biological pathways regulating appetite, nutrient metabolism, energy balance and inflammation [1, 2]. ECs include 2-arachidonoylglycerol (2-AG) and *N*-arachidonoylethanolamide (AEA) [3] while the most studied NAEs are *N*-oleoylethanolamide (OEA), *N*-palmitoylethanolamide (PEA), *N*-linoleoylethanolamide (LEA) and *N*-stearoylethanolamide (SEA) [4, 5].

Circulating NAEs and AEA as well as 2-AG are formed from membrane precursors, such as *N*-acylphosphatidylethanolamines (NAPEs) and diacylglycerol, through the activity of NAPE-specific phospholipase D (NAPE-PLD) and diacylglycerol lipase (DAGL), respectively. Other enzymes hydrolyse NAEs and ECs to fatty acids and ethanolamines [6–8].

ECs elicit their biological activities through the cannabinoid type 1 (CB1) and type 2 (CB2) receptors which are located mainly in the brain but also in peripheral tissues, such as the intestine, liver, skeletal muscle, vascular endothelium, reproductive tissues, and tissues of the immune system [5]. NAEs regulate food intake, glucose homeostasis and inflammation through activation of G protein-coupled receptors (GPCRs), peroxisome proliferator-activated receptors (PPAR-α), and transient receptor vanilloid potential receptors (TRVP) [9–11]. Some of these receptors are located on cells lining in the gastro-intestinal tract (GIT). For example, GPR119 is expressed on cells in the stomach, small intestine and colon [12] and, upon NAE-mediated activation, it elicits the secretion of the insulin-regulating peptides, glucagon-like peptide 1 (GLP-1) and glucose-dependent insulinotropic peptide (GIP) [13, 14]. PPAR-α is expressed in the small intestine, kidneys, liver, heart and brown adipose tissue and increases satiety through its ligand OEA [15–17]. In contrast, 2-AG and AEA stimulate food intake through the CB1 receptors [18].

Mounting evidence in subjects with inflammatory bowel disease and obesity indicates that circulating ECs are key mediators in the interplay between gut, microbiota and metabolic health [19–23]. Moreover, plasma ECs and NAEs are considered biomarkers of white adipose tissue distribution and insulin resistance in obesity [24–32] and, as NAPEs, they are tightly connected with diet, especially dietary fat [1] but also proteins [33, 34].

On the other hand, ECs, NAEs, and NAPEs are present in several foods [35], they increased in saliva upon food mastication in humans [36, 37] and the direct injections of NAPEs in the duodenum reduced food intake in mice [38]. Altogether, this evidence raised the question of whether the luminal content of ECs, NAEs, and NAPEs may be sufficient to elicit biological effects through the receptors located in the GIT.

Therefore, we determined the concentrations of ECs, NAEs and NAPEs in ileal fluids and plasma collected during an observational study in participants with ileostomy and investigated their relationship with habitual macronutrient and energy intake as well as with individual inflammatory status.

## Methods

### Study design

This study is part of an observational study (16/NI/0267) whose primary outcome was the assessment of bone mineral density (by Dual-energy X-ray absorptiometry) in ileostomy patients. Secondary outcomes included: anthropometric measurements, habitual diet, blood lipid profile, blood inflammatory marker, blood polyunsaturated fatty acids, blood and ileal endocannabinoids, and physical activity. The study was conducted with the prior approval of the Office for Research Ethics Committees Northern Ireland (ORECNI), the University of Ulster Ethical Committee and with the informed consent of participants and in accordance with the Declaration of Helsinki. The trial was registered at www.clinicaltrials.gov (NCT04143139). Participants (male and non-pregnant females) recruited to the study were aged 18–70 years and had previously undergone an ileostomy and were 2 + years post-operative at time of recruitment. Participants were excluded if they were outside the desired age range or had their surgical procedure < 2 years before.

Following an overnight fast, the participants visited the clinic of Ulster University in Coleraine (UK) and provided ileal fluid sample and blood samples.

All participants provided questionnaires including a 4-day estimated food diary to establish habitual dietary intake completed within ± 14 days of sampling, subsequently analysed using Nutritics Nutrition Analysis Software. A validated Recent Physical Activity Questionnaire (RPAQ) [39] was also completed by each participant to capture self-reported habitual activity levels at home, work and in leisure time. The total physical activity energy expenditure (PAEE) was estimated by summing up the individual energy expenditure due to each activity domain (home, work and recreational).

### Preparation of biological samples

To avoid handling bias with ileal fluids, we used methodologies consistent with precautions as described in Karu et al*.* [40] for metabolomic analysis in faeces. The ileal fluid samples were collected and processed within 30 min as described in Mc Dougall et al*.* [41]. In brief, volumes and pH values of the ileal fluids were recorded, before dilution with ice-cold distilled water as required, dependent on the viscosity, and before the fluid was homogenized in a chilled Waring blender for 30 s.

Fasting blood samples were collected by venipuncture into serum separator and EDTA-containing tubes. To avoid EDTA blood samples handling bias that may lead to ex vivo biotransformation of monitored compounds, we used recommendations of Gurke and co-workers [42]. All EDTA blood samples were kept chilled/on ice before processing. Plasma samples were prepared by centrifugation at 3000 rpm for 15 min at 4 °C and within 15 min of collection. Serum samples were prepared by allowing blood to clot for 30 min at room temperature, then centrifugation at 3000 rpm for 15 min at 4 °C.

Once prepared, ileal fluids, serum and plasma samples were aliquoted and immediately frozen at − 80 °C. All samples were kept frozen at Ulster University according to Human Tissue Act (HTA) standards until further analysis.

### Extraction of ECs, NAEs and NAPEs from ileal and plasma samples

Simultaneous extractions of ECs, NAEs and NAPEs from ileal fluids and plasma were performed using the method by Bligh and Dyer with brief modifications [43]. All samples were thawed in the fridge at 4 °C before extraction and samples were kept chilled on ice during the specific extraction procedures [42]. The ileal fluids samples were diluted prior the extraction to enhance the efficiency of solvent extraction as previously reported in faeces [40, 44].

Ileal fluids (100 µL) previously diluted 1:10 with distilled water and plasma samples (500 µL) were added to 50 µL of the internal standard 200 ng/mL solution of Arachidonoylethanolamide d8 (AEA d8) (Cayman Chemical, Ann Arbor, MI). A volume of 1.5 mL of CHCl_3_/CH_3_OH (2:1 *v/v*) was added to the sample that was vortexed for 20 s and centrifuged at 14,800 rpm for 10 min at 4 °C. Then, the supernatant was collected in a glass tube and the pellet was extracted with CHCl_3_/CH_3_OH (2:1) twice. KCl 0.07 M (2 mL) was added to the collected phase and the lower layer (chloroform phase) was evaporated under nitrogen flow and reconstituted in 100 µL acetonitrile/isopropanol/water (60:35:5) prior the LC-HRMS analysis.

The extraction recovery was 86% in ileal fluids and 71% in plasma samples.

### Liquid chromatography–high-resolution mass spectrometry (LC-HRMS) analysis

LC-HRMS analysis was performed by adapting the method by Gregory et al*.* [44]. Data were collected using an Accela U-HPLC system consisting of a quaternary pump and a thermostated autosampler (10 °C) coupled to an Exactive Orbitrap MS provided with a heated electrospray interface (HESI) (Thermo Fisher Scientific, San Jose, CA). The compounds were separated on a Kinetex 2.6 μ C18 100 Å column (100 mm × 2.1 mm) (Phenomenex, Torrance, CA) with setting temperature at 45 °C and eluted by a linear gradient of a 40:60 water/acetonitrile mixture (5 mM ammonium formate 0.1% formic acid) (solvent A) and 90:10 isopropanol/acetonitrile (5 mM ammonium formate and 0.1% formic acid) (solvent B) with a flow rate of 200 μL/min and volume injection of 10 μL. According to Gregory et al*.*, eluting gradient was set as follows: 32% B from 0 to 1.5 min, 32–45% B from 1.5 to 4 min, 45–52% B from 4 to 5 min, 52–58% B from 5 to 8 min, 58–66% B from 8 to 11 min, 66–70% B from 11 to 14 min, 70–75% B from 14 to 18 min, 75–97% B from 18 to 21 min and kept at 97% B until 25 min [44]. MS detection was performed in positive- and negative-ion modes in the *m/z* 120–1200 mass range: spray voltage was 3.5 kV (positive mode) and 3.0 kV (negative mode), capillary voltage 30 V, heater temperature 300 °C, capillary temperature at 350 °C, sheath gas 35 and auxiliary gas 15 arbitrary units, respectively.

Compounds were identified and quantified against authentic standards using exact mass value up to the fifth decimal digit (± 5 ppm mass tolerance). ECs (2-AG; AEA; AEAd8) and NAEs (OEA, LEA, PEA) standards were purchased from Cayman (Cayman Chemical, Ann Arbor, MI). *N*-arachidonoylphosphatidylethanolamine standard was purchased from Santa Cruz Biotechnology (Santa Cruz Biotechnology, Inc., USA). SEA was expressed as equivalents of PEA. NAPEs were found in the chromatographic region between 19 and 23 min and were detected in negative-ion mode as [M − H]^−^. In the chromatographic region between 6 and 9 min, the NAEs were detected in positive-ion mode as [M + H]^+^. Supplementary Table 1 reports the molecular formula, theoretical and experimental mass, the mass accuracy and the retention time of identified compounds. The limit of detection (LoD) and limit of quantification (LoQ) of the identified molecules are reported in Supplementary Table 2.

### Biochemical analysis

Circulating lipid profiles (serum triglycerides, total and HDL cholesterol) were quantified on the iLab 650 Clinical Chemistry auto-analyzer (Instrumentation Laboratory, Massachusetts, USA) using a commercially available assay. LDL cholesterol was calculated using the Friedewald formula [45]. Serum C-reactive protein (CRP) was measured at the clinical chemistry department of St James’ Hospital Dublin.

Total lipid was extracted from serum according to a modified version of Folch et al. [46] where chloroform and methanol were used as the extracting solvents in a 2:1 ratio, with 250 µL of plasma extracted in 5 mL of extracting solvents [47], the wash solution was 3:47:48 (chloroform:methanol:water). Fatty acid methyl esters were detected and quantified for six key polyunsaturated fatty acids (PUFA)—linoleic acid (LA), arachidonic acid (AA), α-linolenic acid (ALA), eicosapentaenoic acid (EPA), docosapentaenoic acid (DPA) and docosahexaenoic acid (DHA) using the gold-standard technique of gas chromatography–mass spectrometry (7890A-5975C; Agilent) using heptadecanoic acid (C17:0) as the internal standard, as previously described [47]. In brief, to a 250 µL plasma sample, 18 µL internal standard (heptadecanoic acid (C17:0)) was added and then chloroform:methanol (2:1) to a final volume of 5 mL. The solution was vigorously mixed, 1 mL DDH_2_O added, then centrifuged at 2500*g* for 5 min. The upper layer was discarded, the remaining solution washed twice with chloroform:methanol:water (3:48:47) and the sample filtered, then evaporated to dryness (at 70 °C, under nitrogen). Toluene (100 µL) and borontrifluoride methanol (500 µL) were added and the solution maintained at 100 °C for 1 h. Subsequently, 250 µL of hexane and 800 µL DDH_2_O were added to the cooled sample and mixed. The supernatant was then removed and evaporated to dryness (at 50 °C, under nitrogen). Extractions were reconstituted in 180 µL ethyl acetate before analysis on GC/MS which was completed in split mode (split ratio 1:20), with a BPX70 capillary GC column (SGE Analytical Science) (length 30 m, internal diameter 250 µm and film thickness 0.25 µm), using helium as the carrier gas (constant flow at 1.0 mL/min). Samples were injected using an automatic liquid sampler (ALS) (injection volume 1 µL) at a temperature of 130 °C, this was then ramped at 15 °C/min to 200 °C and then at 30 °C/min to 250 °C where it was held for 5 min. Mass Spectrometry was operated in positive-ion mode using an electron ionisation (EI) source. The mass range was set to 50–500 Da and acquisition was performed by Total Ion Chromatogram (TIC). We identified the individual PUFA—LA, AA, ALA, EPA, DPA and DHA—by their retention time and corresponding qualifier ions with reference to those of commercially available fatty acid standards (Sigma Aldrich, UK). These were quantified by use of an internal standard, heptadecanoic acid (C17:0) (Sigma Aldrich, UK) and corresponding PUFA target ions (quantifiers). For the purpose of this paper, we have defined: Total PUFA as the sum of LA, ALA, AA, EPA, DPA, DHA; n-3 fatty acids as the sum of ALA, EPA, DPA and DHA; n-6 fatty acids as the sum of LA and AA; n-6:n-3 ratio as the ratio between n-6 and n-3 fatty acids.

### Anthropometric measurements

Height and weight were measured using standardized procedures to determine Body Mass Index (BMI, kg/m^2^). Standing height (cm) was measured to the nearest 0.5 cm using a calibrated stadiometer (SECA, Model 220, Germany). Body weight (kg) was recorded without footwear or heavy clothing and was measured to the nearest 0.1 kg using portable scales (Seca; Brosch Direct Ltd, Peterborough, UK). Participants were considered with normal weight when the BMI was in the range 18.5–24.9 kg/m^2^, with overweight when the BMI was in the range 25.0–29.9 kg/m^2^ and with obesity when the BMI was higher than 30 kg/m^2^.

### Statistical analysis

Statistical analyses were performed in R version 3.6.0. After being checked for normality, significantly skewed variables were transformed in ln (*x*) with *k* values. For those that showed a normal distribution at the Kolmogorov–Smirnov test, Student’s *t* test was performed to check differences between sexes. One-way ANOVA and Bonferroni adjustment for multiple comparisons were performed to check differences between compounds in the overall population. For the variables which did not show a normal distribution after logarithmic transformation, a non-parametric Mann–Whitney test was performed. Two-tailed P values lower than 0.05 were considered significantly different. To test correlations between concentrations of NAPEs, NAEs and ECs in biological fluids with the dietary intake, all variables were transformed in ln(*x*) and a Pearson correlation test was performed to check the best model to fit the curve. In all cases, logarithmic model was selected and results obtained were reported. Data are expressed as means ± SEM.

## Results

### Study participants

**Fig. 1:**
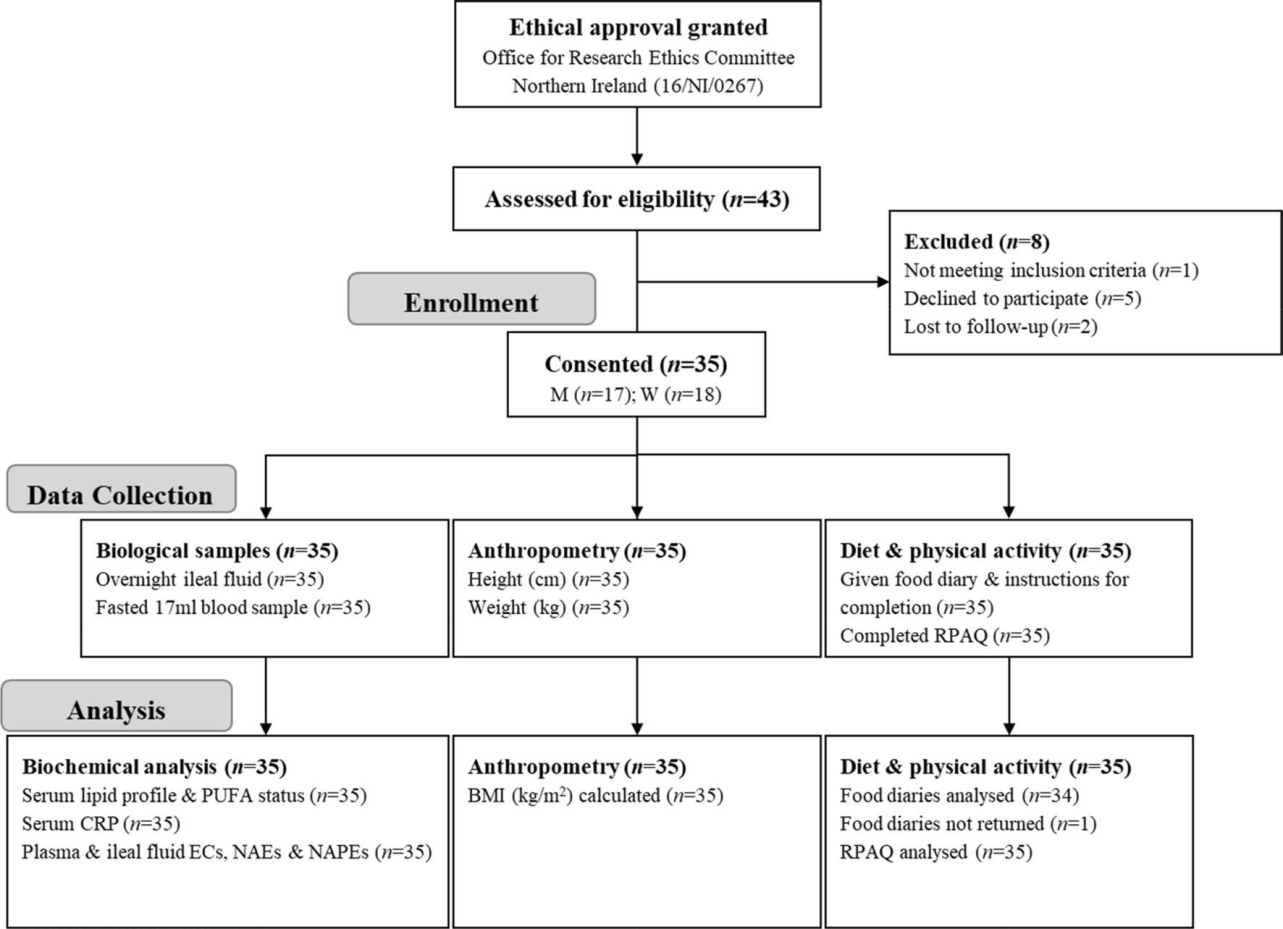
Participants flow

The participant flow is shown in Fig. 1. 35 participants with ileostomy were recruited and completed the study. The characteristics of the participants including general information and anthropometry, serum lipids, PUFA and CRP as well as the mean dosages of each type of medication taken by 27 subjects, are reported in Table 1. Differences between sexes were found for height and weight that were higher, as well as serum HDL-cholesterol concentrations that were lower, in men than women. The participants had a mean BMI of 26.9 ± 0.9 kg/m^2^, specifically there was 1 subject (1 man) who was underweight, 11 subjects (8 women and 3 men) with normal weight (NW), 12 (7 women and 5 men) with overweight (OW) and 11 (3 women and 8 men) with obesity (OB). Compared to NW, OB showed higher triglycerides (1.19 ± 0.15 mmol/L vs 0.6 ± 0.06 mmol/L, *p* = 0.002) and lower HDL-cholesterol concentrations (0.99 ± 0.08 mmol/L vs 1.32 ± 0.06 mmol/L, *p* = 0.013). No significant difference between BMI classes for general characteristics and CRP was observed.

**Table 1:** Characteristics of the study participants with ileostomy

	All (*n* = 35)	Women (*n* = 18)	Men (*n* = 17)	*p* value^a^
General characteristics and anthropometry				
Age (years)	51.1 ± 2.4	50.8 ± 3.8	51.4 ± 3.0	0.898
Height (cm)	168.1 ± 1.3	162.7 ± 1.2	173.9 ± 1.4	** < 0.001**
Weight (kg)	76.4 ± 3.1	68.3 ± 3.1	85.0 ± 4.9	0.006
BMI (kg/m^2^)	26.9 ± 0.9	25.9 ± 1.2	28.0 ± 1.5	0.266
Ileal fluids pH	6.1 ± 0.1	6.1 ± 0.1	6.1 ± 0.1	0.908
Ileal fluids net weight (g)	234.6 ± 15.9	229.1 ± 15.4	240.5 ± 28.9	0.732
PA energy expenditure (MET h/day)^b^	21.6 ± 3.0	16.2 ± 1.8	27.3 ± 5.8	0.087
Serum lipids				
Triglycerides (mmol/L)	0.92 ± 0.08	0.92 ± 0.12	0.92 ± 0.12	0.840
Total cholesterol (TC, mmol/L)	3.52 ± 0.14	3.69 ± 0.19	3.33 ± 0.19	0.190
LDL-cholesterol (mmol/L)	1.94 ± 0.12	2.02 ± 0.17	1.86 ± 0.16	0.509
HDL-cholesterol (mmol/L)	1.16 ± 0.05	1.26 ± 0.04	1.05 ± 0.08	**0.030**
LA (mg/mL)	1.10 ± 0.04	1.16 ± 0.05	1.04 ± 0.05	0.131
ALA (mg/mL)	0.01 ± 0.00	0.01 ± 0.00	0.01 ± 0.00	0.245
AA (mg/mL)	0.28 ± 0.02	0.28 ± 0.02	0.27 ± 0.02	0.815
EPA (mg/mL)	0.01 ± 0.00	0.02 ± 0.00	0.01 ± 0.00	0.226
DPA (mg/mL)	0.01 ± 0.00	0.01 ± 0.00	0.01 ± 0.00	0.918
DHA (mg/mL)	0.03 ± 0.00	0.04 ± 0.01	0.02 ± 0.00	0.093
Total PUFA (mg/mL)^c^	1.45 ± 0.05	1.52 ± 0.07	1.37 ± 0.07	0.144
n-6:n-3 ratio^d^	28.67 ± 3.15	28.41 ± 4.62	28.93 ± 4.40	0.451
Inflammatory status				
CRP (mg/L)	3.30 ± 0.66	3.71 ± 1.18	2.87 ± 0.57	0.719
Medications (mg/kg b.w./day)				
Antihistamine (*n* = 5; 2W, 3M)	0.28 (0.15–0.42)	0.29 (0.15–0.42)	0.27 (0.21–0.35)	
Antidiarrheal (*n* = 5; 2W, 3M)	0.22 (0.08–0.39)	0.17 (0.16–0.17)	0.25 (0.08–0.39)	
Antihypertensive (*n* = 4; 1W, 3M)	1.42 (0.06–3.10)	1.65	1.34 (0.06–3.10)	
Antacids (*n* = 7; 3W, 4M)	0.43 (0.19–0.86)	0.51 (0.42–0.66)	0.38 (0.19–0.86)	
Antidepressant (*n* = 5; 2W, 3M)	0.49 (0.24–1.07)	0.39 (0.32–0.47)	0.56 (0.24–1.07)	
Cholesterol lowering (*n* = 6; 2W, 4M)	0.49 (0.33–0.66)	0.62 (0.58–0.66)	0.42 (0.33–0.51)	

*BMI* Body mass index, *PA* physical activity, *LA* linoleic acid, *ALA* alpha-linolenic acid, *AA* arachidonic acid, *EPA* eicosapentaenoic acid, *DPA* docosapentaenoic acid, *DHA* docosahexaenoic acid, *PUFA* polyunsaturated fatty acids, *CRP* C-reactive protein

Data are expressed as means ± SEM. Medications are expressed as means (range) of dosages (mg/kg body weight/day) taken by total (*n*) subjects; *n* women (W), *n* men (M)

^a^*p* < 0.05 in bold indicates significant difference between women and men, by Student’s *t* test or Mann-Whithney test depending on data normal distribution

^b^Self-reported data collected using a validated physical activity (PA) questionnaire [39]

^c^Total PUFA: sum of LA, ALA, AA, EPA, DPA, DHA

^d^n-6:n-3 ratio: (sum of LA and AA)/(sum of ALA, EPA, DPA and DHA)

### Concentration of ECs, NAEs and NAPEs in ileal fluids

**Fig. 2:**
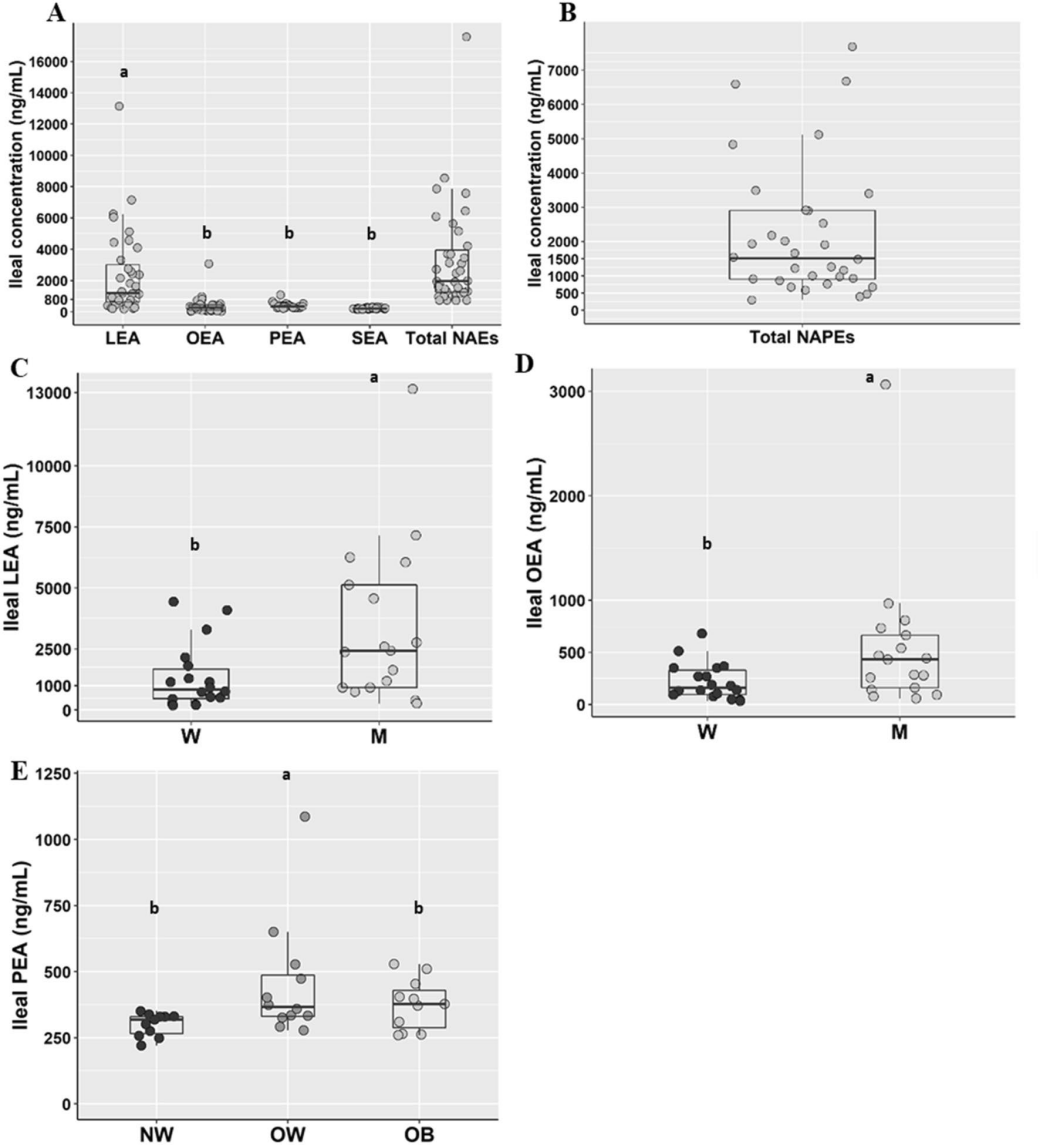
*N*-acylethanolamines (**a**) and *N*-acylphosphatidylethanolamines (**b**) concentrations in ileal fluids from the overall population (*n* = 35), LEA (**c**) and OEA (**d**) concentrations in ileal fluids from men (M, empty dots; *n* = 17) and women (W, solid dots; *n* = 18) and PEA (**e**) concentrations in ileal fluids from participants with normalweight (NW; *n* = 11), overweight (OW; *n* = 12) and obesity. Different letters on the box plots indicate *p* value < 0.05 by One-way ANOVA and Bonferroni adjustement for multiple comparisons or by Student’s *t* test. Total NAEs include the sum of LEA, OEA, PEA and SEA. *LEA* Linoylethanolamide, *OEA* oleoylethanolamide, *PEA* palmitoylethanolamide, *SEA* stearoylethanolamide, *NAEs N*-acylethanolammines, *NAPEs N*-acylphosphatidylethanolamines. The box plots show the data distribution based on first quartile, median and third quartile

Figure 2 shows the ileal concentrations of monitored NAEs and NAPEs in overall population and some significant differences between sexes and BMI classes.

Mean concentrations of NAEs and NAPEs were not significantly different (3346.94 ± 561.39 ng/mL vs 4896.99 ± 2043.01 ng/mL).

LEA was the most abundant NAE and it was, like OEA, significantly higher in men than women while PEA concentration was significantly higher in OW than NW.

No differences between sexes in ileal concentrations of PEA, SEA and NAPEs or between BMI classes in ileal LEA, OEA, SEA and NAPEs concentrations were found (Supplementary Fig. 1).

### Plasma concentration of ECs, NAEs and NAPEs

**Fig. 3:**
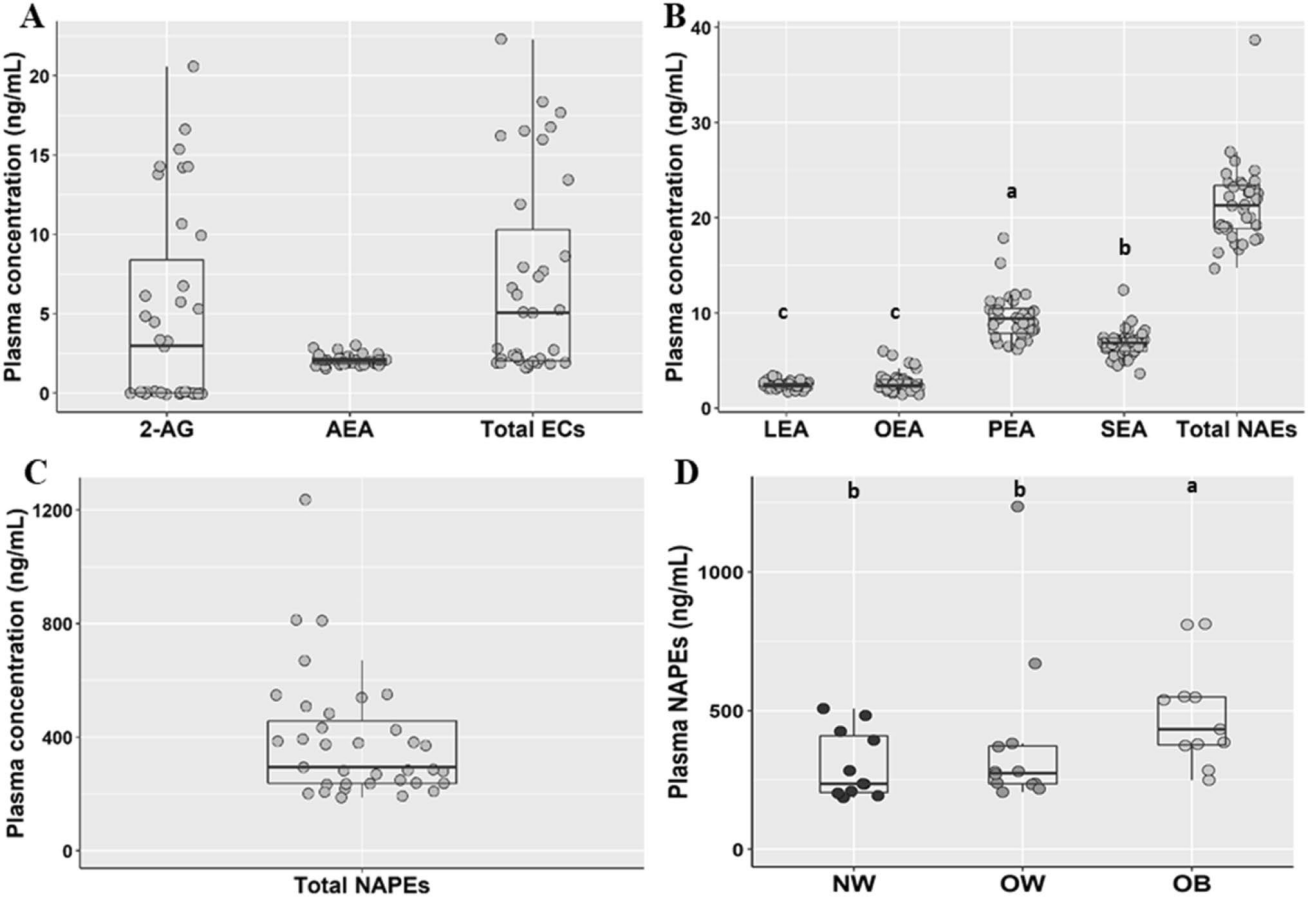
Plasma Endocannabinoids (**a**), *N*-acylethanolammines (**b**) and *N*-acylphosphatidylethanolamines (**c**) concentrations in the overall population (*n* = 35) and *N*-acylphosphatidylethanolamines (**d**) plasma concentrations from participants with normalweight (NW; *n* = 11), overweight (OW; *n* = 12) and obesity (OB, *n* = 11). Different letters on the box plots indicate *p* value < 0.05 by One-way ANOVA and Bonferroni adjustement for multiple comparisons. Total ECs include the sum of 2-AG and AEA; total NAEs include the sum of LEA, OEA, PEA and SEA. *2-AG* 2-Arachidonoylglicerol, *AEA* arachidonoylethanolamide, *ECs* endocannabinoids, *LEA* linoylethanolamide, *OEA* oleoylethanolamide, *PEA* palmitoylethanolamide, *SEA* stearoylethanolamide, *NAEs N*-acylethanolammines, *NAPEs N*-acylphosphatidylethanolamines. The box plots show the data distribution based on first quartile, median and third quartile

Figure 3 shows the plasma concentrations of all monitored compounds in the population. In contrast to ileal fluids, AEA and 2-AG were frequently detected and in similar concentrations in plasma. PEA was the most abundant NAE followed by SEA, and similar concentration of OEA and LEA.

No difference in plasma NAEs between sexes and between BMI classes was observed (Supplementary Fig. 2). Contrarily, circulating levels of plasma NAPEs were higher in OB compared to NW subjects (Fig. 3).

**Fig. 4:**
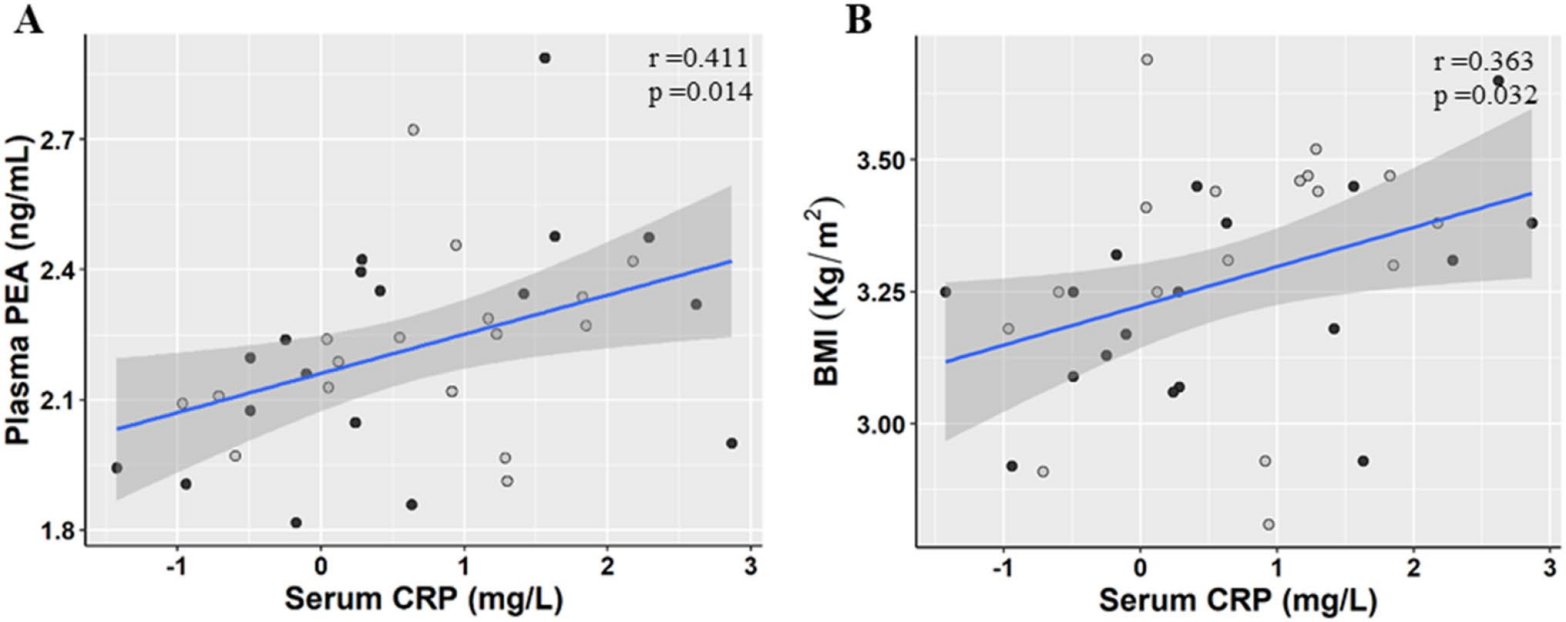
Correlation between individual plasma concentrations of PEA (**a**), BMI (**b**) and individual serum CRP. Men (M, *n* = 17) are indicated with empty dots and women (W, *n* = 18) with solid dots. R and *p* value are assessed by Pearson correlation on ln transformed variables. *PEA* palmitoylethanolamide, *CRP* C-reactive protein, *BMI* Body Mass Index

Interestingly, positive logarithmic correlations between plasma CRP with PEA (*r* = 0.411, *p* = 0.014) and BMI (*r* = 0.363, *p* = 0.032) were found (Fig. 4). Although, subjects who took supplements had lower serum CRP concentrations than those who did not (2.66 ± 0.95 and 3.83 ± 0.92, *p* = 0.024) (Supplementary Fig. 3), supplement consumption did not affect plasma ECs, NAEs and NAPEs concentration.

No significant association between plasma concentration of the monitored compounds and age with the exception of a negative association between AEA plasma and age in men (*r* = − 0.522, *p* = 0.032) independently of BMI, was found.

### Diet and correlations with plasma and ileal fluids of ECs, NAEs and NAPEs

Table 2 shows daily energy value and macronutrient composition of diets self-reported by the participants. Women reported significantly lower energy, carbohydrate, protein and fat intakes than men but a similar repartition of energy among macronutrients; only the % energy from dietary fiber was higher in women than in men. Self-reported dietary intakes and plasma concentrations of PUFAs were similar between sexes (Table 1). Overall dietary intake of n-3 PUFAs was lower (1.2 ± 0.21 g) compared to the intake of n-6 PUFAs (5.8 ± 0.68 g), reflecting a limited consumption of foods like oily fish in the cohort with only 6 out of 34 participants reporting intake (group average 55 ± 60 g/day) and only two consumed canned oily fish (group average 23.1 ± 9.7 g/day). Whereas foods rich in n-6 PUFAs, such as chicken (22/34 participants, group average 65.7 ± 61.2 g/day) and eggs (20/34 participants, group average 21.8 g ± 12.1 g/day), were consumed in greater quantities and by more participants. Only nine participants consumed nuts and seed (group average 17.6 ± 16.0 g/day) a rich source of MUFAs. The relative amounts (g) of self-reported food consumption for all participants in the study are shown in Supplementary Table 3.

**Table 2:** Nutritional composition of habitual diets of all the study participants and by sexes

	All (*n* = 34)	Women (*n* = 18)	Men (*n* = 16)	*p* value^a^
Energy (kJ kcal)	8292.8 ± 364.2	7176.5 ± 393.7	9548.7 ± 473.4	** < 0.001**
	1982.0 ± 87.1	1715.2 ± 94.1	2282.2 ± 113.1	
Carbohydrates (g)	221.9 ± 9.3	195.8 ± 9.9	251.3 ± 13.0	**0.002**
% Energy	45.3 ± 1.0	46.2 ± 1.4	44.3 ± 1.4	0.334
Dietary fiber (g)	17.8 ± 0.8	17.7 ± 1.2	17.9 ± 1.0	0.914
% Energy	1.9 ± 0.1	2.1 ± 0.2	1.6 ± 0.1	**0.006**
Proteins (g)	85.5 ± 4.6	73.0 ± 5.4	99.5 ± 6.1	**0.002**
% Energy	17.4 ± 0.7	17.0 ± 0.8	17.9 ± 1.2	0.519
Fats (g)	79.7 ± 5.0	67.2 ± 5.4	93.7 ± 7.4	**0.006**
% Energy	35.4 ± 1.1	34.7 ± 1.6	36.2 ± 1.4	0.476
SFA (g)	30.2 ± 2.5	24.6 ± 2.7	36.5 ± 4.0	**0.011**
MUFA (g)	29.0 ± 1.9	23.9 ± 2.0	34.8 ± 2.6	**0.002**
PUFA (g)	12.4 ± 0.9	11.0 ± 1.1	13.9 ± 1.5	0.107
Total n-3 FA (g)	1.2 ± 0.2	1.0 ± 0.2	1.4 ± 0.4	0.325
Total n-6 FA (g)	5.8 ± 0.7	6.3 ± 1.0	5.3 ± 1.0	0.458
Trans fats (g)	1.1 ± 0.1	1.0 ± 0.2	1.1 ± 0.1	0.584

Data are expressed as mean ± SEM, obtained from self-reported 4-day food diaries

*SFA* saturated fatty acids, *MUFA* monounsaturated fatty acids, *PUFA* polyunsaturated fatty acids, *n-3 FA* omega-3 fatty acids, *n-6 FA* omega-6 fatty acids

^a^*p* < 0.05 in bold indicates significant difference between women and men, by Student’s *t* test

A logarithmic correlation analysis between ileal and plasma concentrations of ECs, NAEs and NAPEs with habitual self-reported energy and nutrient intakes, was performed. Results showed that ileal concentrations of LEA positively correlated with individual energy intake whereas plasma concentrations of LEA, OEA and AEA inversely correlated with it. Moreover, we found that ileal concentrations of LEA and OEA positively correlated with fat intake whereas plasma concentrations of LEA, OEA, and AEA were negatively correlated. Specifically, plasma concentrations of LEA and AEA negatively correlated with saturated fat intake; PEA negatively correlated with n6, n3 series and PUFA intake; OEA negatively correlated with PUFA and AEA with MUFA intake. Moreover ileal LEA and OEA were positively associated with saturated fat and trans fat intake. All the correlations found are detailed in Supplementary Table 4.

### Correlations between serum PUFA and ECs, NAEs and NAPEs

Plasma LEA and 2-AG concentrations were negatively associated with AA while SEA was positively associated with DHA and n3 fatty acids. Concentrations of NAPEs in ileal fluids were positively associated with plasma EPA and DPA. All the correlations found are shown in Supplementary Table 5.

## Discussion

The implications of the endocannabinoid system and its role in human health have become an area of increasing interest over the last decade [5]. To date, only a limited number of studies have actually measured ECs, NAEs and NAPEs in human biological fluids with circulating ECs reported in the range 0.2–9.4 ng/mL (12 studies), NAEs ranging 0.5–64.6 ng/mL (10 studies), and NAPEs ranging 120.7–800.0 ng/mL (2 studies) [29, 33, 34, 48–50]. While ECs are acknowledged key mediators in the interplay between gut, microbiota and metabolic health, to the best of our knowledge, their levels and those of NAEs and NAPEs in the gastro-intestinal tract are largely unknown with only one recent human study reporting fecal concentration for single NAPE [51]. In this study, for the first time, the concentrations of ECs, NAEs and NAPEs within the GIT are measured, via ileal fluid, revealing the amount of the compounds that would be colon-available upon leaving the small intestine. We determined that ileal NAEs and particularly OEA (383.72 ± 88.78 ng/mL), LEA (2360.10 ± 454.74 ng/mL) and PEA (377.72 ± 26.08 ng/mL) were on average 1.5–13 times higher than that required to elicit a physiological response through the intestinal receptors. Indeed, the agonist activity (EC50) of such compounds on GPR119 ranges from 65 ng/mL to 1.0 µg/mL for OEA, 180 ng/mL for LEA and 250 ng/mL for PEA [52, 53]; OEA also displayed agonist activity on PPAR-α with an EC50 of 39 ng/mL [15].

In the present study, ileal samples were collected from stoma bags filled during the overnight fasting period and consequently may contain some food residues from the last meal consumed by the participants prior to the fasting period. Therefore, food eaten in that meal might have directly influenced the lipid type and amount in ileal fluids whose composition is more susceptible to individual digestion rate than fasting plasma samples. This may explain the differences in the lipids’ profiles between ileal fluids and plasma and why, for example, LEA is the most abundant NAE in the ileal samples while it is the less abundant in plasma. Indeed, we hypothesize that dietary NAEs and NAPEs [35] may result from digestive processes in the proximal GIT following mastication [36, 37], being into the alimentary canal postprandially at concentrations even higher than those found in this study. Such an effect could trigger GLP-1 secretion by enterocytes through activation of intestinal GPR119, thus contributing to the homeostatic regulation of food intake, which is consistent with the findings from Chen and co-workers [54] showing that feeding mice with NAPE-synthesizing bacteria increased delivery of NAEs/NAPEs in the intestinal lumen and significantly reduced food intake and adiposity induced by a high-fat diet. In the case of intestinal NAPEs, their activity necessitates conversion into NAEs through the action of intestinal NAPE-hydrolyzing phospholipase D [55]. NAEs and NAPEs, therefore, appear to be more effective in eliciting a leptogenic effect from the intestinal lumen than in other tissues. Consequently, more robustly designed studies are needed to elucidate the physiological relevance of diet-induced increases in intestinal NAEs and NAPEs postprandially.

It should be noted, that correlations between ileal and plasma LEA and OEA with energy and fat intakes concomitant with the sex differences suggested that an individual’s diet may influence NAEs intestinal and plasma concentrations. Dosoky and co-workers reported that fecal concentrations of NAPEs (NAEs precursors) in mice fed with a plant-based diet were significantly higher than in mice fed a lard-based high-fat diet [56], which is in line with our observations that NAPEs are more abundant in plant compared to animal foods [35].

In agreement with Balvers and co-workers, we found that total plasmatic NAPEs were about 13- to 32-fold higher than NAEs [57] and were more abundant in OB than NW and OW. Plasma concentrations of ECs and NAEs in the ileostomy cohort (average BMI 26.9 ± 0.9) were in the same order of magnitude to those previously measured in overweight and obese subjects with intact GI tracts [19, 28, 29, 32]. In contrast, we did not find any association between 2-AG and AEA with BMI and this is consistent with the reported heterogeneity of observations in the literature, whereby plasma 2-AG has been positively correlated with BMI [25, 26] and yet no in other studies [19, 58]. Similarly, AEA plasma concentrations were elevated in obese subjects compared to normal weight subjects [19, 27, 32] yet other studies report no association with BMI [25, 26].

Other characteristics than BMI which are often disregarded in the populations studied, such as the body composition, the number of women in menopause or the use of drugs or dietary supplements, may be responsible of the different findings between literature studies.

We found sex correlations between the plasma concentrations of AEA, OEA and PEA with individual age and only our observations in women were in agreement with Fanelli et al*.* [32]. However, serum-free fatty acids were not positively associated with NAEs and AEA in contrast to a previous study in healthy women [59]. The heterogeneity observed is likely due in part to the different age and BMI ranges of the study populations used.

Few studies currently in the literature, reported on whether drugs affected plasma ECs and NAEs other than in relation to treating depression, whereby circulating 2-AG was halved while AEA was unchanged in patients with major depression compared to healthy subjects [60, 61] and antidepressant use did not change plasma EC nor NAE concentrations [61]. In our study, AEA plasma concentrations were about 2- to 4-fold higher than those of depressed patients [60, 61], whereas 2-AG plasma concentrations were consistent [61] or ~ 15-fold lower than patients with depression [60].

Inflammatory status (CRP) was positively correlated with the nutritional status as assessed by BMI and was consistent with previous studies [62]. On the other hand, the positive association we found between plasma concentrations of PEA and CRP, independently of BMI, was in agreement with animal and human studies showing that circulating and tissue PEA concentrations increased in subjects suffering from inflammatory diseases, such as neuropathic and inflammatory pain or ulcerative colitis [63]. Furthermore, a recent study reported elevated concentrations of a NAPE, namely 18:1/16:1/18:0, in the faeces of patients suffering from ulcerative colitis in comparison to healthy controls [51].

This study has some limitations. Firstly, the habitual diet and physical activity energy expenditure were estimated by self-reported data from participants using previously validated survey instruments, which is not as reliable as direct observations. Second, since we did not measure total energy expenditure, we could not estimate if participants were in energy balance. If subjects were not in energy balance, this might alter the implications of our observations. Finally, the possible presence of residues of the dinner in the ileal fluids makes any attempt to identify the relationships between ileal and plasma content challenging in this study.

In conclusion, we assessed, for the first time, the concentrations of ECs, NAEs and NAPEs present in ileal fluids from participants with ileostomy and, thus, established the amounts likely to be entering the colon in individuals with an intact GIT. Men had higher ileal concentrations of OEA and LEA than women which positively correlated with the self-reported dietary higher energy and fat intake. An ongoing intervention study will address whether the dietary intakes of NAPEs and NAEs affect the concentrations of NAEs in the intestinal lumen postprandially and if they contribute to the homeostatic control of food intake.

## Electronic supplementary material

Below is the link to the electronic supplementary material.

Supplementary file1 (DOCX 510 kb)

Supplementary file2 (XLSX 26 kb)

## Abbreviations

2-AG: 2-Arachidonylglycerol
AA: Arachidonic acid
AEA: Anandamide
AEAd8: Anandamide-d8
ALA: α-Linolenic acid
CB1: Cannabinoid receptor-1
CRP: C-reactive protein
DHA: Docosaexaenoic acid
DPA: Docosapentaenoic acid
ECs: Endocannabinoids
EPA: Eicosapentaenoic acid
FAAH: Fatty acids amide hydrolases
GIP: Glucose-dependent insulinotropic polypeptide
GIT: Gastrointestinal tract
GLP-1: Glucagon-like peptide 1
GPCRs: G-protein-coupled receptors
LA: Linoleic acid
LC-HRMS: Liquid chromatography–high-resolution mass spectrometry
LEA: Linoleoylethanolamide
MUFA: Monounsaturated fatty acids
NAAA: *N*-Acylethanolamine-hydrolyzing acid amidase
NAEs: *N*-Acylethanolamines
NAPE-PLD: *N*-Acyl-phosphatidylethanolamine-specific phospholipase D
NAPEs: *N*-Acylphosphatidylethanolamines
OEA: Oleoylethanolamide
PEA: Palmitoylethanolamide
PAEE: Physical activity energy expenditure
PPAR-α: Peroxisome proliferator-activated receptor
PUFA: Polyunsaturated fatty acids
RPAQ: Recent physical activity questionnaire
SFA: Saturated fatty acids
