# Supplementary material for: Endocannabinoids, endocannabinoid-like molecules and their precursors in human small intestinal lumen and plasma: does diet affect them?
Source: Eur J Nutr. 2020 Oct 26;60(4):2203–15. doi: 10.1007/s00394-020-02398-8 (PMC8137602; doi:10.1007/s00394-020-02398-8)
Supplement: Supplementary file 1 — Supplementary file1 (DOCX 510 kb) [file 394_2020_2398_MOESM1_ESM.docx]

**Electronic Supplementary Material**

**Endocannabinoids, endocannabinoid-like molecules and their precursors in human small intestinal lumen and plasma: does diet affect them?**

*European Journal of Nutrition*

Silvia Tagliamonte, Chris I.R. Gill, L. Kirsty Pourshahidi, Mary M. Slevin, Rosalia Ferracane, Roger Lawther, Gloria O’Connor, Paola Vitaglione*****

***Corresponding author:**

Prof Paola Vitaglione,

University of Naples “Federico II”,

Via Università 100, 80055 Portici (NA) Italy;

E-mail: [*paola.vitaglione@unina.it*](mailto:paola.vitaglione@unina.it)

**Supplementary Table 1.** ECs, NAEs and NAPEs identification by High-Resolution Mass Spectrometry.

| Compound^a^ | Molecular formula | Theoretical mass  [M+H]^+^ *m/z* [M-H]^-^ *m/z* | | Experimental  mass | Mass accuracy (ppm) |
| --- | --- | --- | --- | --- | --- |
| ECs |  |  |  |  |  |
| AEA | C_22_H_37_NO_2_ | 348.28971 |  | 348.28877 | -2.70 |
| 2-AG | C_23_H_38_O_4_ | 379.28429 |  | 379.28436 | 0.18 |
| AEA-d8 | C_22_H_29_D_8_NO_2_ | 356.33992 |  | 356.33987 | -0.14 |
| NAEs |  |  |  |  |  |
| OEA | C_20_H_39_NO_2_ | 326.30536 |  | 326.30499 | -1.13 |
| LEA | C_20_H_37_NO_2_ | 324.28971 |  | 324.28995 | 0.74 |
| PEA | C_18_H_37_NO_2_ | 300.28971 |  | 300.28976 | 0.17 |
| SEA | C_20_H_41_NO_2_ | 328.32101 |  | 328.32098 | -0.09 |
| NAPEs |  |  |  |  |  |
|  | C_55_H_104_NO_9_P |  | 952.73759 | 952.73984 | 2.36 |
|  | C_57_H_104_NO_9_P |  | 976.73759 | 976.73890 | 1.34 |
|  | C_57_H_106_NO_9_P |  | 978.75324 | 978.75163 | -1.64 |
|  | C_59_H_104_NO_9_P |  | 1000.73759 | 1000.74099 | 3.40 |
|  | C_59_H_106_NO_9_P |  | 1002.75324 | 1002.75533 | 2.08 |
|  | C_59_H_102_NO_9_P |  | 998.72194 | 998.72536 | 3.42 |
|  | C_59_H_108_NO_9_P |  | 1004.76889 | 1004.77163 | 2.73 |
|  | C_61_H_112_NO_9_P |  | 1032.80019 | 1032.80093 | 0.72 |
|  | C_57_H_108_NO_9_P |  | 980.76889 | 980.77113 | 2.28 |
|  | C_59_H_110_NO_9_P |  | 1006.78454 | 1006.78540 | 0.85 |
|  | C_59_H_112_NO_9_P |  | 1008.80019 | 1008.80127 | 1.07 |
|  | C_61_H_110_NO_9_P |  | 1030.78454 | 1030.78635 | 1.76 |
|  | C_61_H_108_NO_9_P |  | 1028.76889 | 1028.77166 | 2.69 |
|  | C_65_H_108_NO_9_P |  | 1076.76889 | 1076.76953 | 0.59 |
|  | C_58_H_11_0NO_9_P |  | 994.78454 | 994.78229 | -2.26 |
|  | C_63_H_108_NO_9_P |  | 1052.76889 | 1052.77161 | 2.58 |
|  | C_61_H_104_NO_9_P |  | 1024.73759 | 1024.73608 | -1.47 |

^a^2-AG, 2-Arachidonoylglicerol; AEA, Arachidonoylethanolamide; AEA-d8, Arachidonoylethanolamide d8; ECs, Endocannabinoids; LEA, Linoylethanolamide; NAEs, *N*-acylethanolammines; NAPEs, *N*-acylphosphatidylethanolamine; OEA, Oleoylethanolamide; PEA, Palmitoylethanolamide; SEA, Stearoylethanolamide.

**Supplementary Table 2.** ECs, NAEs and NAPEs limit of detection (LoD) and limit of quantitation (LoQ).

| Compound ^a^ | LoD (ng/mL) | LoQ (ng/mL) |
| --- | --- | --- |
| Endocannabinoids |  |  |
| AEA | 0.1 | 0.25 |
| 2-AG | 1 | 2.5 |
| N-acylethanolamines |  |  |
| OEA | 0.1 | 0.25 |
| LEA | 0.1 | 0.25 |
| PEA | 0.1 | 0.25 |
| N-Arachidonoylphosphatidylethanolamine | 100 | 200 |

^a^ 2-AG, 2-Arachidonoylglicerol; AEA, Arachidonoylethanolamide; ECs, Endocannabinoids; LEA, Linoylethanolamide; NAEs, *N*-acylethanolammines; NAPEs, *N*-acylphosphatidylethanolamines; OEA, Oleoylethanolamide; PEA, Palmitoylethanolamide.

**Supplementary Table 3.** [**https://drive.google.com/file/d/1c3--QPXyU6R03BHS7f7BXQ6rhzlPVDIb/view?usp=sharing**](https://drive.google.com/file/d/1c3--QPXyU6R03BHS7f7BXQ6rhzlPVDIb/view?usp=sharing)

**Supplementary Table 4.** Logarithmic correlations between individual reported energy, protein, carbohydrate, fat intake, SFA, MUFA, PUFA, n-6, n-3 and trans fats intake with plasma and ileal concentrations of LEA, OEA, PEA, SEA, AEA, 2-AG, NAPEs.^a^

|  | | **Energy intake** | **Protein**  **intake** | **Carbohydrates intake** | **Fat**  **intake** | **SFA**  **intake** | **MUFA intake** | **PUFA intake** | **n-6 FA intake** | **n-3 FA intake** | **Trans**  **fats intake** |
| --- | --- | --- | --- | --- | --- | --- | --- | --- | --- | --- | --- |
| **Plasma LEA** | **r** | -0.364* | -0.296 | -0.320 | -0.353* | -0.364* | -0.336 | 0.001 | 0.046 | 0.199 | -0.282 |
|  | **p-value** | **0.034** | 0.089 | 0.065 | **0.041** | **0.034** | 0.052 | 0.994 | 0.796 | 0.259 | 0.106 |
| **Plasma PEA** | **r** | -0.228 | -0.208 | -0.124 | -0.300 | -0.153 | -0.279 | -0.493** | -0.377* | -0.394* | -0.219 |
|  | **p-value** | 0.195 | 0.237 | 0.486 | 0.084 | 0.388 | 0.110 | **0.003** | **0.028** | **0.021** | 0.214 |
| **Plasma OEA** | **r** | -0.363* | -0.305 | -0.275 | -0.434* | -0.276 | -0.400* | -0.402* | -0.290 | -0.191 | -0.183 |
|  | **p-value** | **0.035** | 0.080 | 0.116 | **0.010** | 0.115 | 0.019 | **0.018** | 0.097 | 0.278 | 0.300 |
| **Plasma SEA** | **r** | 0.101 | 0.055 | 0.184 | 0.000 | 0.022 | 0.02 | -0.139 | -0.234 | -0.09 | -0.097 |
|  | **p-value** | 0.569 | 0.759 | 0.297 | 0.999 | 0.903 | 0.912 | 0.432 | 0.183 | 0.612 | 0.586 |
| **Plasma AEA** | **r** | -0.385* | -0.261 | -0.321 | -0.472** | -0.422* | -0.438** | -0.201 | -0.145 | -0.07 | -0.278 |
|  | **p-value** | **0.025** | 0.136 | 0.065 | **0.005** | **0.013** | **0.009** | 0.255 | 0.414 | 0.693 | 0.111 |
| **Plasma 2-AG** | **r** | -0.051 | -0.135 | -0.076 | 0.094 | -0.064 | 0.006 | 0.185 | 0.190 | 0.274 | -0.124 |
|  | **p-value** | 0.775 | 0.447 | 0.669 | 0.595 | 0.720 | 0.973 | 0.296 | 0.282 | 0.117 | 0.485 |
| **Plasma NAPEs** | **r** | -0.031 | 0.032 | 0.003 | -0.079 | 0.007 | -0.083 | -0.017 | -0.176 | -0.228 | 0.082 |
|  | **p-value** | 0.862 | 0.858 | 0.985 | 0.656 | 0.968 | 0.640 | 0.924 | 0.320 | 0.195 | 0.645 |
| **Ileal LEA** | **r** | 0.378^*^ | 0.332 | 0.248 | 0.401^*^ | 0.508** | 0.311 | 0.076 | -0.03 | 0.099 | 0.347* |
|  | **p-value** | **0.028** | 0.055 | 0.157 | **0.019** | **0.002** | 0.074 | 0.669 | 0.866 | 0.576 | **0.044** |
| **Ileal PEA** | **r** | 0.103 | 0.270 | 0.023 | 0.080 | 0.333 | 0.094 | -0.135 | -0.178 | 0.005 | 0.250 |
|  | **p-value** | 0.563 | 0.122 | 0.898 | 0.653 | 0.054 | 0.598 | 0.446 | 0.313 | 0.975 | 0.153 |
| **Ileal OEA** | **r** | 0.323 | 0.285 | 0.204 | 0.369^*^ | 0.498** | 0.281 | 0.029 | -0.08 | -0.003 | 0.403* |
|  | **p-value** | 0.062 | 0.103 | 0.246 | **0.032** | **0.003** | 0.107 | 0.870 | 0.654 | 0.989 | **0.018** |
| **Ileal SEA** | **r** | -0.176 | 0.102 | -0.257 | -0.223 | -0.099 | -0.174 | -0.182 | -0.201 | -0.074 | -0.122 |
|  | **p-value** | 0.319 | 0.567 | 0.142 | 0.205 | 0.578 | 0.325 | 0.302 | 0.254 | 0.679 | 0.490 |
| **Ileal NAPEs** | **r** | 0.275 | 0.263 | 0.278 | 0.271 | 0.111 | -0.036 | -0.03 | -0.02 | 0.043 | 0.064 |
|  | **p-value** | 0.115 | 0.133 | 0.112 | 0.121 | 0.531 | 0.839 | 0.868 | 0.910 | 0.810 | 0.720 |

^a^ Data are r and p-values assessed by Pearson correlation (n=35). 2-AG, 2-Arachidonoylglicerol; AEA, Arachidonoylethanolamide; ECs, Endocannabinoids; LEA, Linoylethanolamide; MUFA, Monounsaturated fatty acids; NAEs, *N*-acylethanolammines; NAPEs, *N*-acylphosphatidylethanolamine; n-3 FA, Omega-3 fatty acids; n-6 FA, Omega-6 fatty acids; OEA, Oleoylethanolamide; PEA, Palmitoylethanolamide; PUFA, Polyunsaturated fatty acids; SEA, Stearoylethanolamide; SFA, Saturated fatty acids; * p-value<0.05; **p-value<0.01.

**Supplementary Table 5.** Correlation between serum LA, ALA, AA, EPA, DPA, DHA, Total PUFA, n-3 FA, n-6 FA, and n-6:n-3 ratio levels with LEA, OEA, PEA, SEA, AEA, 2-AG, NAPEs in plasma and ileal fluids. ^a^

|  | | **LA** | **ALA** | **AA** | **EPA** | **DPA** | **DHA** | **Total PUFA** | **n-3 FA** | **n-6 FA** | **n-6:n-3 ratio** |
| --- | --- | --- | --- | --- | --- | --- | --- | --- | --- | --- | --- |
| **Plasma LEA** | **r** | 0.045 | -0.282 | -0.354^*^ | -0.229 | -0.310 | 0.001 | -0.109 | -0.200 | -0.092 | 0.044 |
|  | **p-value** | 0.798 | 0.100 | **0.037** | 0.185 | 0.070 | 0.994 | 0.533 | 0.249 | 0.600 | 0.804 |
| **Plasma PEA** | **r** | 0.018 | 0.089 | -0.083 | 0.058 | -0.112 | 0.070 | -0.008 | 0.034 | -0.015 | 0.040 |
|  | **p-value** | 0.918 | 0.611 | 0.634 | 0.742 | 0.523 | 0.691 | 0.964 | 0.847 | 0.932 | 0.824 |
| **Plasma OEA** | **r** | 0.061 | -0.138 | -0.177 | 0.111 | -0.158 | 0.317 | 0.012 | 0.156 | -0.013 | -0.211 |
|  | **p-value** | 0.728 | 0.430 | 0.309 | 0.526 | 0.366 | 0.064 | 0.946 | 0.370 | 0.941 | 0.231 |
| **Plasma SEA** | **r** | 0.225 | 0.044 | 0.144 | 0.258 | 0.275 | 0.356^*^ | 0.273 | 0.368^*^ | 0.243 | -0.206 |
|  | **p-value** | 0.194 | 0.801 | 0.411 | 0.134 | 0.110 | **0.036** | 0.113 | **0.030** | 0.160 | 0.241 |
| **Plasma AEA** | **r** | -0.042 | -0.276 | -0.100 | -0.080 | -0.325 | -0.039 | -0.089 | -0.172 | -0.074 | -0.016 |
|  | **p-value** | 0.810 | 0.108 | 0.566 | 0.649 | 0.057 | 0.826 | 0.610 | 0.324 | 0.671 | 0.928 |
| **Plasma 2-AG** | **r** | -0.063 | -0.220 | -0.540^**^ | -0.215 | -0.180 | -0.092 | -0.250 | -0.200 | -0.246 | 0.047 |
|  | **p-value** | 0.718 | 0.204 | **0.001** | 0.215 | 0.300 | 0.599 | 0.147 | 0.248 | 0.154 | 0.792 |
| **Plasma NAPEs** | **r** | -0.177 | 0.023 | 0.299 | 0.332 | 0.231 | 0.057 | -0.010 | 0.230 | -0.042 | -0.179 |
|  | **p-value** | 0.310 | 0.897 | 0.081 | 0.051 | 0.183 | 0.745 | 0.954 | 0.184 | 0.813 | 0.311 |
| **Ileal LEA** | **r** | 0.251 | -0.007 | 0.039 | 0.054 | 0.172 | -0.054 | 0.211 | 0.030 | 0.227 | 0.001 |
|  | **p-value** | 0.145 | 0.968 | 0.826 | 0.759 | 0.322 | 0.758 | 0.223 | 0.863 | 0.191 | 0.994 |
| **Ileal PEA** | **r** | 0.235 | -0.049 | 0.106 | 0.164 | 0.181 | -0.031 | 0.226 | 0.082 | 0.236 | -0.064 |
|  | **p-value** | 0.175 | 0.782 | 0.543 | 0.346 | 0.298 | 0.860 | 0.191 | 0.639 | 0.172 | 0.718 |
| **Ileal OEA** | **r** | 0.285 | 0.054 | 0.005 | 0.086 | 0.154 | 0.026 | 0.233 | 0.082 | 0.244 | -0.065 |
|  | **p-value** | 0.097 | 0.756 | 0.978 | 0.624 | 0.378 | 0.882 | 0.178 | 0.641 | 0.158 | 0.714 |
| **Ileal SEA** | **r** | 0.084 | -0.074 | 0.270 | 0.176 | 0.122 | -0.062 | 0.157 | 0.066 | 0.166 | -0.017 |
|  | **p-value** | 0.630 | 0.671 | 0.117 | 0.313 | 0.484 | 0.725 | 0.369 | 0.708 | 0.340 | 0.924 |
| **Ileal NAPEs** | **r** | -0.127 | -0.022 | 0.323 | 0.364^*^ | 0.398^*^ | 0.012 | 0.043 | 0.260 | 0.008 | -0.195 |
|  | **p-value** | 0.469 | 0.898 | 0.058 | **0.032** | **0.018** | 0.945 | 0.805 | 0.132 | 0.964 | 0.269 |

^a^ Data are r and p-values assessed by Pearson correlation (n=35). LA, Linoleic acid; ALA, α-linolenic acid; AA, Arachidonic acid; EPA, Eicosapentaenoic acid; DPA, Docosapentaenoic acid; DHA, Docosahexaenoic acid; Total PUFA, Polyunsaturated fatty acids as sum of LA, ALA, AA, EPA, DPA, DHA; n-3 FA, sum of ALA, EPA, DPA and DHA; n-6 FA, sum of LA and AA; n-6:n-3 ratio: n-6 FA/n-3 FA; 2-AG, 2-Arachidonoylglicerol; AEA, Arachidonoylethanolamide; ECs, Endocannabinoids; LEA, Linoylethanolamide; NAEs, *N*-acylethanolammines; NAPEs, *N*-acylphosphatidylethanolamine; OEA, Oleoylethanolamide; PEA, Palmitoylethanolamide; SEA, Stearoylethanolamide; * p-value<0.05; **p-value<0.01.


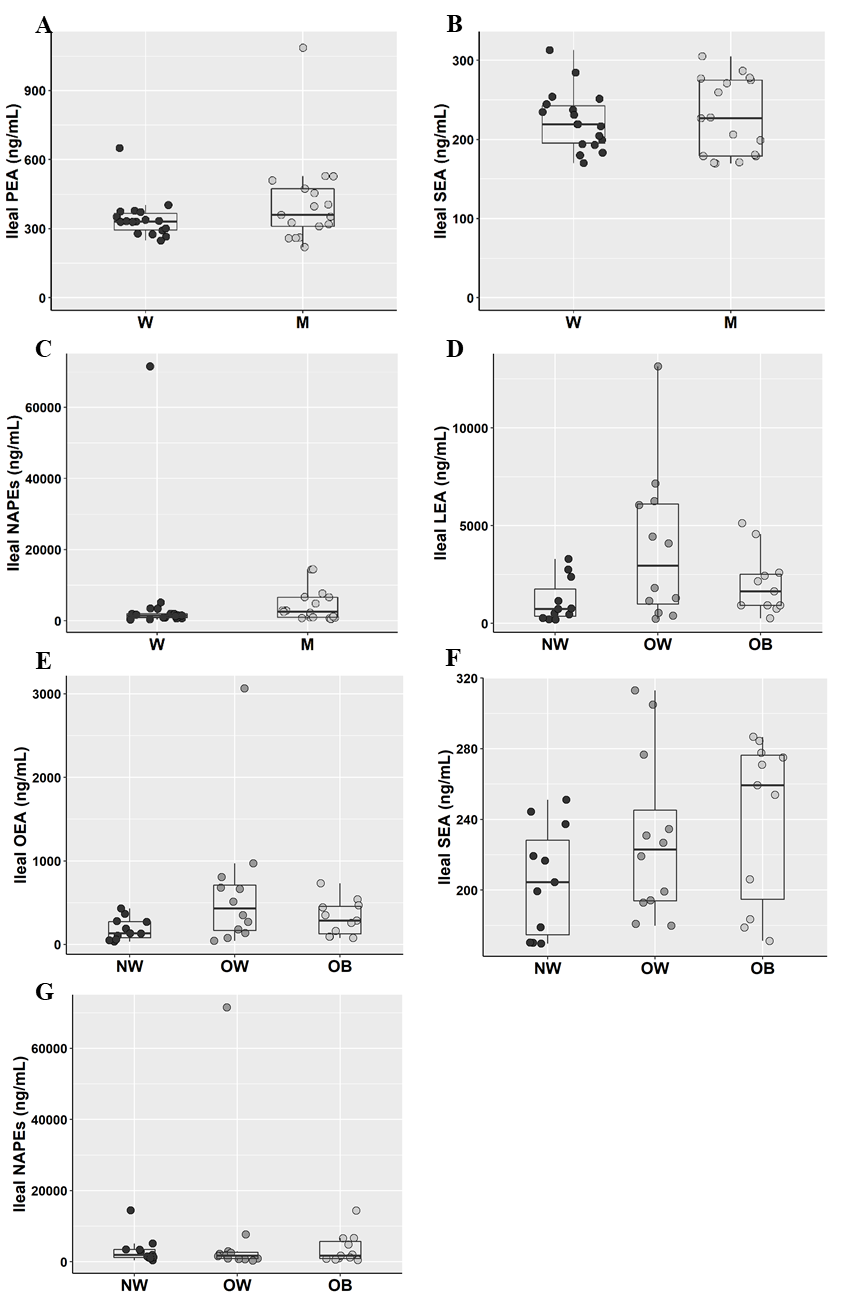


**Supplementary Fig. 1:** Ileal concentrations of PEA (A), SEA (B) and NAPEs (C) in men (M, empty dots; n=17) and women (W, solid dots; n=18) and ileal concentrations of LEA (D), OEA (E), SEA (F), NAPEs (G) in participants with normalweight (NW; n=11), overweight (OW; n=12) and obesity (OB, n=11). PEA, Palmitoylethanolamide; SEA, Stearoylethanolamide; LEA, Linoylethanolamide; OEA, Oleoylethanolamide; NAPEs, *N*-acylphosphatidylethanolamines. The box plots show the data distribution based on first quartile, median and third quartile.

Data showed no significant difference between sexes (by Student’s t test) in ileal concentrations of PEA, SEA and NAPEs or between BMI classes (by One-way ANOVA) in ileal concentrations of LEA, OEA, SEA and NAPEs.


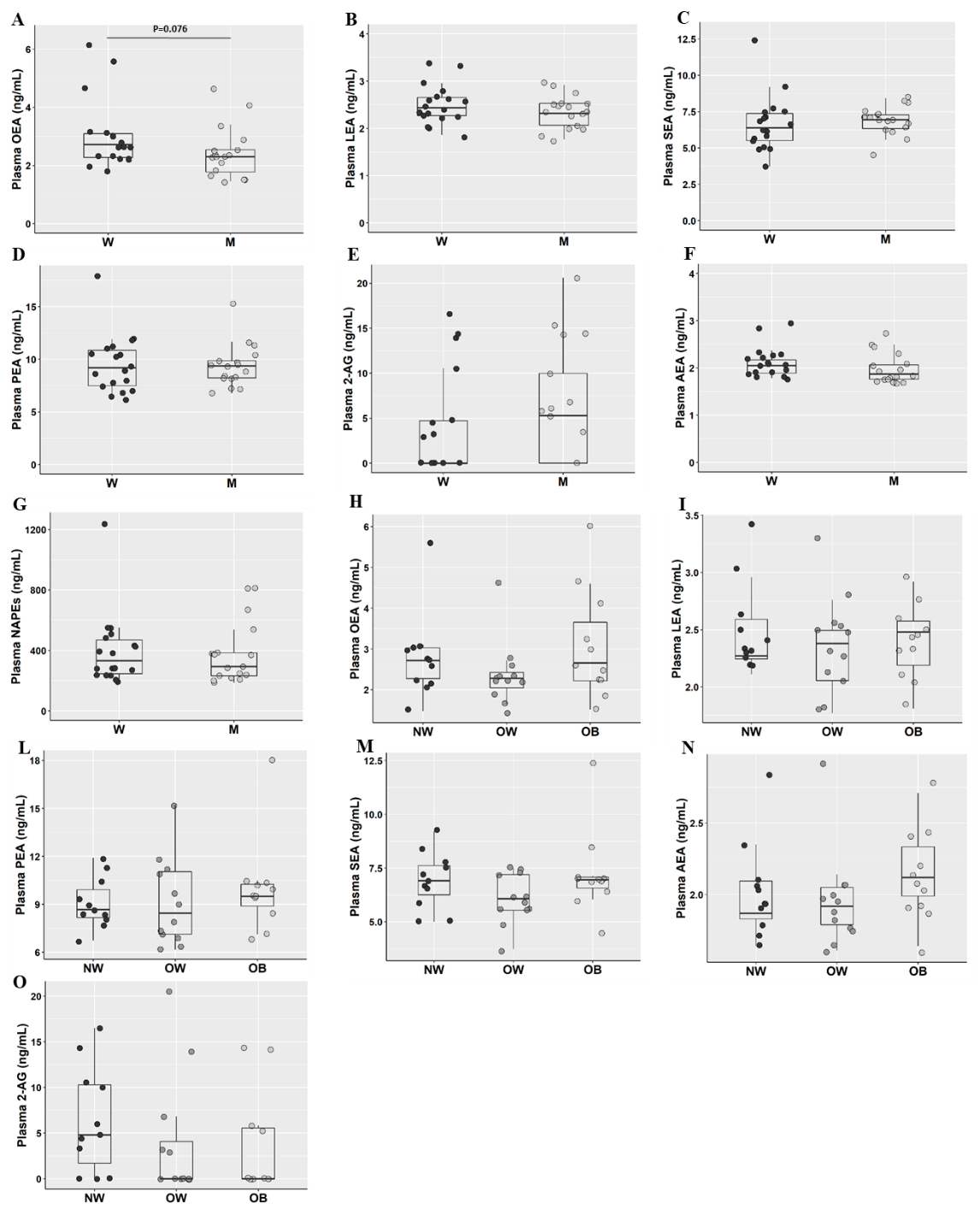


**Supplementary Fig. 2:** Plasma OEA (A)**,** Plasma LEA (B), SEA (C), PEA (D), 2-AG (E), AEA (F), and NAPEs (G) concentrations in men (M, empty dots; n=17) and women (W, solid dots; n=18) and ileal concentrations of OEA (H), LEA (I), PEA (L), SEA (M), AEA (N) and 2-AG (O) in participants with normalweight (NW; n=11), overweight (OW; n=12) and obesity (OB, n=11). LEA, Linoylethanolamide; PEA, Palmitoylethanolamide; OEA, Oleoylethanolamide; SEA, Stearoylethanolamide; AEA, Arachidonoylethanolamide; 2-AG, 2-Arachidonoylglicerol; NAPEs, *N*-acylphosphatidylethanolamines. The box plots show the data distribution based on first quartile, median and third quartile. Plasma OEA concentration showed a trend towards higher concentrations in W than M (p<0.1 by Student’s t test). No difference or trend (p>0.1) between W and M (by Student’s t test) or BMI classes (by One-way ANOVA) for the other monitored compounds was found.


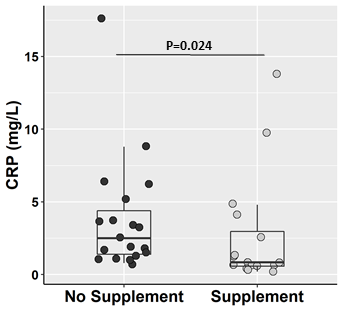


**Supplementary Fig. 3:** Serum concentrations of CRP in subject taking supplements (empty dots; n=16) and those who are not taking supplemnts (solid dots; n=19). P-value was assessed by Mann-Whitney test. CRP, C-reactive protein. The box plots show the data distribution based on first quartile, median and third quartile.
